# Pathology and solutions for activities of medical education development offices

**DOI:** 10.1186/s12909-023-05011-8

**Published:** 2024-01-03

**Authors:** Parvin Rezaei Gazki, Somayeh Noori Hekmat, Mahla Salajegheh

**Affiliations:** 1 Department of Medical Education, Medical Education Development Center, Kerman University of Medical Sciences, Kerman, Iran; 2 Health Services Management Research Center, Institute for Futures Studies in Health, Kerman University of Medical Sciences, Kerman, Iran

**Keywords:** Medical education, Management, Organization, Education Development Center (EDC), Education Development Office (EDO)

## Abstract

**Background and aim:**

Education development offices are one of the main branches of medical education centers for directing the educational performance of medical sciences universities to achieve educational goals. Due to their close presence and communication with educational environments, these offices are highly important. To effectively guide and empower these offices, it is necessary to analyze their current situation, identify the challenges, and provide solutions to address them. This study was conducted to identify the challenges and provide solutions for the activities of medical education development offices.

**Methods:**

This qualitative study was conducted in two stages, including 29 semi-structured interviews and a focus group discussion with experts in 2022 at Kerman University of Medical Sciences. The sampling method was purposive. The content analysis of data was performed based on conventional qualitative content analysis.

**Results:**

Data analysis resulted in the emergence of two main categories including challenges facing the activities of medical education development offices and solutions for improving the activities of these offices, and comprising some categories containing organizational structure factors, cognitive factors, communication factors, and motivational factors.

**Conclusion:**

Education development offices are one of medical universities’ main policymaking and quality control institutions. Efforts are being made to establish EDOs structures within the university. The formation of a clear and performance-based reward system for faculty members who are the managers of the EDOs is proposed. Improving interactions between EDOs and other parts of the university to coordinate activities, and exchange of experiences are highlighted.

## Introduction

The developments of healthcare organizations, the increase in the complexity of healthcare, and the changing concepts of medical education have revealed the need for transformation and change in medical education more than ever before [1]. This has led to the establishment of education development centers (EDCs) aimed at improving the quality of educational services. These centers are responsible for decision-making and policy implementation regarding the improvement of the quality of education in universities [2]. The World Health Organization states that the mission of EDCs is to collect and classify information related to education, quality, and quantity of human resources required for health and medical services [3]. In Iran, the establishment of EDCs was put on the agenda after the founding of such a center in the educational department of the Ministry of Health, Treatment, and Medical Education in late 1989. The aim of establishing these centers is to improve the quality of medical education [4]. As enhancing the quality of education is one of the main goals of medical education worldwide [5], and it is also one of the policies of our country’s development plans in higher education, there have been many studies on the performance of education development centers in Iran.

A review of these studies demonstrates the importance and necessity of the existence and activities of EDCs, as well as the wide-ranging expectations of faculty members from these centers [2–6]. These results indicate the significance and sensitivity of the role of EDCs in improving the quality of education in medical sciences universities [2]. However, despite the emphasis on the high-quality performance of EDCs, in many cases, the current performance status of these centers is not considered desirable by many stakeholders in educational services [3, 7, 8]. Recommendations for improvement include the need to enhance the performance of education development offices (EDOs) and support them [2].

Education development offices that located in schools and hospitals in medical sciences universities are the main arms of EDCs in achieving educational goals. These offices are highly important due to their close presence and connection with educational environments. To extend educational development activities to all educational sectors of universities, EDOs have been established as executors of quality improvement activities in schools and hospitals [9]. According to the regulations of EDCs and EDOs, their roles include monitoring and supervising the educational performance of schools/hospitals, empowering faculty members, supporting faculty members in implementing innovative educational and research projects, creating student development committees in schools/ hospitals, and being involved in educational planning [10].

Despite the extensive role of these offices in developing educational quality, they face challenges such as being far from EDC, limited foundational knowledge of collaborating faculty members in these offices regarding medical education, their multiple organizational roles, and simultaneous occupations of faculty members in schools and hospitals. In spite of the importance of the existence, structure, and performance of EDOs in achieving educational goals, research on the challenges faced by these offices and finding appropriate solutions is limited. Keshmiri and colleague (2020) evaluated the performance of EDOs at an average level. They emphasized the need for complete familiarity of EDOs with their duties and roles, as well as the growth of developmental infrastructures and the creation of managerial support to fulfill their assigned tasks in the university [11]. Heydari et al. (2021) evaluated the EDOs and revealed that the overall performance of these offices is at an average level. They highlighted that to meet the evolving needs and keep up with the latest developments in medical education, EDOs must continuously enhance themselves and become sources of educational innovations [12]. Taghavinia and colleague (2020) recognized the performance criteria of EDCs and enlightened on the necessity of evaluating the performance of EDOs to improve the performance and the quality of medical education and enhance community health [13]. Faghihi et al. (2020) investigated key educational and research factors affecting the future of the medical education discipline and discovered that EDOs have a significant role in this regard [14].

Given the crucial role of EDOs in improving the quality of education in universities, continuous monitoring of these offices can play an important role in improving their performance and accelerating education improvement processes. However, systematic planning to evaluate the performance of EDOs in medical sciences universities has received less attention from educational managers. Therefore, analyzing their current situation, identifying their problems, and providing strategies to address them are essential for effective guidance and empowerment of these offices. This information will lead to the presentation of solutions aimed at improving performance and accelerating the process of educational development, as well as effective guidance and empowerment of EDOs. Therefore, this study was conducted to identify the challenges and provide solutions for the activities of medical education development offices.

## Methods

### Study design

This study has a descriptive qualitative design [15]. Data was collected through semi-structured interviews and expert opinions were examined using a focus group discussion. Data was analyzed using conventional content analysis [16]. This methodology was chosen as a means of gaining rich insight into the participants’ experiences. This study was conducted in 2022 at the Kerman University of Medical Sciences.

### Setting and participants

Kerman University of Medical Sciences (KMU) is the 8th largest medical university in Iran and the largest university of medical sciences in the southeast of the country with about 6000 students and 700 faculty members. This university has 13 EDOs in 8 schools including Medicine, Dentistry, Pharmacy, Nursing and Midwifery, Public Health, Paramedics and Allied Medicine, Iranian Traditional Medicine and Health Care Management and Information, and 5 educational hospitals.

The purposive sampling method was maximum variation which emphasized on significant shared patterns that cut across cases and derived their important from having emerged out of heterogeneity [17]. In this regard, the participants were 29 individuals including managers of EDOs, employees of EDOs, faculty members with experience in EDOs, and managers of EDCs in other medical sciences universities. In selecting the sample, efforts were made to choose individuals with diverse organizational roles related to EDOs and EDCs.

The inclusion criteria included being interested in participating in the study, faculty members with responsibility or experience in EDOs, and employees serving in EDOs. The exclusion criteria were unwillingness to participate, conduct or continue in the study.

### Data collection

The research was conducted in two stages: 1) semi-structured interviews and 2) expert opinions were examined using a focus group discussion.

To conduct the interviews, first, questions and an interview guide were developed by reviewing similar studies. Then, after coordinating with each participant, the research objectives, participation rights, and refusal to participate in the interviews were explained to them. After obtaining informed consent, the interviews were conducted using the interview guide (Table 1). During the interviews, the entire content was recorded with the participant’s consent. Throughout the interviews, continuous efforts were made to provide sufficient opportunity for the participants to express their opinions and avoid providing information that might bias the responses. Sampling continued until data saturation was reached. The average duration of the interviews was 45 min.

**Table 1:** The interview guide

**Questions:**
1. My team and I are trying to understand what is your experience with the challenges of the activities of medical education development offices and what do you suggest for the solutions?
2. From your lens, describe the obstacles because of these challenges to your school or hospital. Share one of your observations or personal experience.
3. Please elaborate on your perceptions of how solutions can have organizational results (or are not).
4. Based on your experience, what are the factors that led to these results? What factors can increase these outcomes?
5. What do you suggest to overcome these obstacles?
6. If you were in the place of managers, what solution would you take to face the existing challenges?
7. Anything else you would like to share about the challenges and solutions for the activities of medical education development offices?
**Probe for:**
a) Challenges/solutions/activities of medical education development offices

Regards the complex and multidimensional nature of educational activities which involves a multifaceted combination of attitudes, resources, strategies, and skills, and usually deals with complex human activities, a focus group discussion was conducted to provide a deeper understanding of the challenges and solutions for the activities of medical education development offices. In this method, it supposed that collaboration within the groups would offer richer data than individual interviews, by generating a synergy where shared point of views would result to an innovative and deeper perception of the research subject [18].

To conducting focus group, an electronic invitation was sent to attend in focus group for 5 faculty members with responsibility or experience in EDOs and 6 specialized faculty members in medical education. The purpose of inviting was explained in the invitation. In the focus group discussion, consent was obtained again from the participants. In this meeting, the opinions of 11 experts on the challenges and solutions of the activities of EDOs were collected. The session lasted about an hour and a half. The session leader attempted to engage all participants in the discussion and allowed everyone to share their experiences and opinions. All discussions were recorded, and notes were taken on the discussions and points.

### Data analysis

The interviews and the data from the focus group discussion were transcribed verbatim and then analyzed using conventional content analysis which borrows from inductive content analysis [16]. Content analysis is a systematic method for analyzing qualitative data that enlightens similarities within and differences between the data. It allows the analysis of descriptive and interpretative data that leads in categories [16].

An inductive approach, which involved immersing the researcher in the data was applied to search for patterns in the texts and contained a series of steps [19]. First, one author (PR) read the transcribed texts several times to gain an initial overview of the data. Any interesting quotes were marked, and notes/comments were made in the margin. The second step involved de-contextualizing the data by extracting quotes from the transcribed texts, so-called meaning units, condensing them without altering their meaning, and then assigning them codes [20]. PR and MS grouped similar codes into 22 sub-categories and 8 categories that represent the manifest content. This process was not linear but involved discussion between the authors and movement back and forth between the different parts and the text as a whole. The third step included a process of reflection and discussion on the underlying meaning in the categories to abstract and interpret latent content in the data and go beyond the participants’ actual words. In all of this process, the research members tried to have regular team meetings to facilitate co-construction of findings and resolve differences in interpretation of data and careful documentation of decisions made throughout the study design, data collection, and analysis to establish confirmability and dependability.

### Rigor and trustworthiness

To assess the rigor and trustworthiness of the findings, four criteria presented by Guba and Lincoln were used, including credibility, dependability or consistency, transferability, and confirmability [21]. In this study, various methods were used to confirm the data validity, such as long-term involvement with data, purposeful sampling, concurrent data collection and analysis, immediate implementation of interview transcripts, and review by colleagues. Also, a summary of the transcripts was returned to the participants as a member check and approved by them. To confirm the dependability criterion, the data were rigorously reviewed by an external auditor. Since one of the authors was in a position of authority, it was significant to ensure participants did not feel forced. Her role as a researcher in this setting was carefully described, and one of the co-authors aided at each interview to strengthen rigor. To confirm the transferability criterion, the researchers provided a detailed and rich description of the participant’s background and characteristics, study context, and clear explanation of limitations and obstacles, and conditions for using the findings in other contexts. To meet the confirmability criterion, all research stages, especially data analysis, were documented in detail so that other researchers could easily follow up on the study based on the available interviews, analyses, and other research stages documentation. The rigor in focus group discussion was enhanced through flexible systematic sampling, ensuring participants had the freedom to state their opinions, ensuring accurate transcription and data-driven coding, and ongoing attention to context [22].

### Ethical considerations

This research was approved by the Research Ethics Committee of Kerman University of Medical Sciences (IR.KMU.REC.1402.255). Participants did not receive any incentives, and participation was voluntary. Informed verbal and written consent for participation was obtained based on the proposal approved by the ethics committee. The participants were also assured of the confidentiality of their information, and it was explained that the results would only be used for research objectives.

## Results

The results were obtained through analyzing 29 interviews and analyzing the data collected from the focus group discussion. Eleven interviews were conducted with faculty members who were responsible for EDOs in KMU, ten interviews with employees of EDOs, and eight interviews with managers of EDCs. The demographic characteristics of the interviewees and experts who participated in the focus group discussion are presented in Tables 2 and 3.

**Table 2:** Demographic characteristics of the interviewees

Interviewees	Gender		Academic Rank		Total
	Female	Male	Assistant professors	Associate professors	
Managers of EDOs	8 (72/7%)	3 (27/7%)	10 (9/09%)	1 (90/9%)	11
Managers of EDCs	4 (50%)	4 (50%)	4 (50%)	4 (50%)	8
Employees of EDOs	8 (80%)	2 (20%)	-	-	10

**Table 3:** Demographic characteristics of experts in the focus group discussion

	Gender		Academic Rank		Total
	Female	Male	Assistant professors	Associate professors	
Experts in focus group discussion	8 (72/7%)	3 (27/7%)	7 (63/6%)	4 (36/3%)	11

The qualitative data analysis led to the emergence of 2 main categories, 8 categories, and 22 sub-subcategories (Table 4). These findings were the result of categorizing 170 primary codes.

**Table 4:** Results of data analysis

Main categories	Categories	Sub-categories
Challenges of EDOs	Organizational structure factors	Absence of EDOs
		Inactivity of EDOs
		Lack of supply
		Lack of transparency in the organizational hierarchy
		Lack of information regarding the importance and philosophy of EDOs
		Administrative bureaucracies
	Cognitive factors	Lack of sufficient knowledge
	Communication factors	Insufficient communication
	Motivational factors	Lack of self-confidence
		Lack of motivation in managers of EDOs
		Lack of motivation in faculty members to participate in EDOs
Solutions for improving the performance of EDOs	Organizational structure solutions	Establishment of EDOs
		Activating EDOs
		Provision of organizational resources
		Clarification of organizational hierarchy
		Reduction of administrative bureaucracies
		Monitoring the activities of EDOs
	Cognitive solutions	Strengthening specialized knowledge
	Communication solutions	Facilitating the communication of EDOs with the decision-making aspect of the university
	Motivational solutions	Strengthening the confidence of managers of EDOs
		Motivating managers of EDOs
		Motivating faculty members

### Challenges of EDOs

This main category refers to the challenges that EDOs face and includes 4 subcategories: organizational structure factors, cognitive factors, communication factors, and motivational factors.

#### Organizational structure factors

This subcategory includes problems that are mainly related to the lack or insufficient presence of EDOs in the organizational structure of school or hospital. It includes 6 sub-subcategories: absence of EDOs, inactivity of EDOs, lack of supply, lack of transparency in the organizational hierarchy, lack of information regarding the importance and philosophy of EDOs, and administrative bureaucracies.

According to the participants, in some cases, there may not be a place for the establishment and operation of EDOs in the organizational structure of the university, which can have a negative impact on the quality of education. One of the faculty members stated:

“In all schools, there are EDOs, but in a small school like ours, this office has not been formed at all, and we practically do not have any external presence” (Interviewee 3)

Some interviewees believed that despite the existence of EDOs, these offices are inactive in practice.

“EDO meetings were better held before COVID-19; now there is no news about them. They are simply closed.” (Interviewee 5)

Some participants considered resource shortages (human, financial, equipment, facilities, etc.) as important factors affecting the poor performance of EDOs.

“We do not have an employee in our office. We have to collect information ourselves, which is very troublesome” (Interviewee 4)

From the perspective of some participants, lack of transparency in the organizational hierarchy is one of the factors that lead to challenges in the performance of EDOs.

“It is not clear whether the EDOs are under the supervision of the deputy of education or the EDC” (Interviewee 3)

Some participants believed that the lack of information in some managers of the schools/hospitals, some managers of EDCs, and even employees regarding the importance and existence philosophy of EDOs creates challenges in their performance.

“The EDC is doing its own job; I think it’s redundant to have an EDO to do the same thing” (Participant 8 in focus group discussion)

“Faculty do not come to us; they go straight to the EDC” (Interviewee 2)

Some participants in this study found the administrative process for conducting the activities of EDOs to be complicated.

“With all this paperwork and numerous systems, colleagues have no interest or motivation for activities in education. It is better to conduct research in our own fields and write an article relevant to our field” (Interviewee 1)

#### Cognitive factors

This category includes challenges related to cognitive factors in the attitudes of colleagues in EDOs. It includes a subcategory of insufficient knowledge. According to the participants, in some cases, insufficient knowledge about medical education and educational management among the managers and employees in EDOs can be a significant challenge to the functioning of these offices.

“We did not study this activity at all. Their language is heavy, and it is not understandable for us” (Interviewee 4)

#### Communication factors

This category addresses the impact of formal and informal communications as a significant challenge for the operation of EDOs. It includes a subcategory of insufficient communication.

Some participants believed that insufficient communication between some EDOs and the decision-making body of the school could have a negative impact on the performance of these offices due to reasons such as lack of executive power and limited work experience of the faculty members who are responsible for these offices.

“The EDOs do not have much executive power. We don’t even know our colleagues. We need more power than we have” (Interviewee 6)

“Our communication with the educational deputy is not strong enough; in any school where the educational deputy is involved, things go well” (Participant 3 in focus group discussion)

#### Motivational factors

This category includes three subcategories, including lack of sufficient self-confidence, lack of motivation among managers of EDOs, and lack of motivation among faculty members to participate in these offices. It focuses on motivational deficiencies.

Some participants considered the lack of sufficient self-confidence among managers of EDOs to engage in activities related to the mission of these offices as one of the effective challenges.

“Workshop instructors should come from EDC rather than EDOs. Even if we study and resolve issues, we can’t get into this area.” (Participant 2 in focus group discussion)

From the perspective of some participants in the study, the lack of motivation among some managers of EDOs and the lack of motivation among faculty members to participate in activities related to educational development has negative effects on the performance of these offices.

“There is no incentive for colleagues in practice. Even if it exists in the regulations, it is not implemented in practice.” (Interviewee 4)

“Faculty members are not familiar with EDOs at all, and they have no interest in joining their committees.” (Interviewee 5)

### Solutions for improving the performance of EDOs

This main category is divided into four subcategories, including organizational structure solutions, cognitive solutions, communication solutions, and motivational solutions.

#### Organizational structure solutions

This subcategory includes solutions that mainly relate to strengthening the organizational structure in schools and hospitals to improve the performance of EDOs. It includes six subcategories, including establishment of EDOs, activating EDOs, provision of organizational resources, clarification of organizational hierarchy, reduction of administrative bureaucracies, and monitoring the activities of EDOs.

According to the participants, in some cases, creating or enabling EDOs in small schools or hospitals can be very helpful. To this end, developing specific plans and operational plans for the EDOs can help. Also, determining the responsibility of managers of those EDOs who do not have enough presence or willingness to continue their activities should be considered. Additionally, following up on activity reports and following up on the resolutions adopted at meetings can be helpful. Some participants also suggested holding explanatory meetings with new managers and employees to review last year’s performance report and provide feedback.

“The issuance of communications should be pursued for faculty members who agree to serve as managers of the development committee.” (Interviewee 1)

“It is good for EDOs to hold regular meetings.” (Interviewee 5)

From the perspective of some participants in the study, providing human resources, especially full-time or part-time employees in EDOs, is very useful for improving the performance of these offices. Clarifying organizational hierarchies and how organizational communication works simultaneously with the EDC, the educational deputy of the university, justifying the importance and philosophy of the existence of EDOs can also be very effective.

“That part of the meeting where you explained the new regulations of the EDOs duties clarified many issues.” (Participant 4 in focus group discussion)

Some participants stated that developing a monitoring program for the activities of EDOs to supervise their activities can improve their performance.

#### Cognitive solutions

This subcategory includes solutions that aim to strengthen the knowledge of managers and employees in EDOs to improve their performance. Strengthening their scientific basis in medical education, holding in-service training for development office employees, and providing useful specialized consultation are all helpful. Empowering EDOs to independently hold faculty development programs and guiding them in research proposals for education is also effective. One faculty member stated:

“It is good to provide a short training on medical education at the beginning of monthly meetings by the managers of EDOs.” (Interviewee 8)

#### Communication solutions

This category comprises strategies that facilitate communication between EDOs and decision-making bodies of schools, resulting in improved performance of the EDOs. Based on participant feedback, solutions such as regularly holding meetings between EDOs and EDC, and establishing communication between EDOs and educational councils of universities/hospitals to cover the necessary network for interventions in universities/hospitals can be utilized.

One interviewee mentioned that their school has the resources to hold one of the faculty development sessions: “We can hold one of the empowerment sessions in our school next month.” (Interviewee 7).

#### Motivational solutions

Based on participant feedback, building confidence in EDOs managers to engage in activities related to EDOs can be a key motivator. This can be achieved through engaging them in teaching or collaborating in faculty development programs. Motivating them can also be accomplished by actively evaluating the performance of EDOs and allocating organizational reinforcements to active EDOs while focusing more on activities related to developing the quality of education. Additionally, creating motivation among faculty members to participate in activities related to educational development can be accomplished by introducing them to the activities of EDC and EDOs. In this regard, one interviewee mentioned that the orientation tour provided for newly hired faculty members was very helpful.

## Discussion

Based on the results of this study, the challenges of the activities in the EDOs and their corresponding solutions were categorized into four main categories: organizational structure factors, cognitive factors, communication factors, and motivational factors.

According to the findings, organizational structure challenges include the lack of EDOs, inactive EDOs, resource shortages, lack of transparency in the organizational hierarchy, inadequate justification of the importance and philosophy of the existence of EDOs, and administrative bureaucracies.

Some of the challenges faced by EDOs activities relate to the overall structure of the organization as well as the organizational position of these offices. When the organizational chart of a hospital does not include a designated position for EDOs, managers will be less motivated to establish or operate a dedicated unit. On the other hand, these offices may not know which part of the hospital or university they are considered subordinate to and from which part they should receive organizational directives. Therefore, in some hospitals, these units are directly under the presidency, while in other places, they are under the Deputy for Education, and they are unaware of the organizational connection with the university’s EDC. Many decision-makers are not familiar with the mission and organizational responsibilities of these offices, which is why they do not make efforts to address organizational issues. Even some managers in these offices mistakenly confuse their duties with those of the Continuous Medical Education Center. Additionally, bureaucracies, parallel work processes, and organizational resource constraints, both financial and non-financial, are other challenges that overshadow these offices.

Organizational challenges refer to challenges that can exist in all organizations regardless of the individuality of employees and their private or governmental work environment. These challenges can be considered as one of the main challenges of organizational productivity.

Regarding organizational structure challenges, in line with this study, Karimi et al. (2015) also highlighted organizational factors as influential factors on organizational productivity [23]. In Sohrabi et al. (2019) study, among the three categories of individual, environmental, and organizational factors, organizational barriers had the greatest impact on reducing productivity [24]. Arab Mokhtari et al. (2020) revealed that organizational culture had a more important role in explaining productivity compared to other factors [25]. Additionally, Dehnavieh et al. (2019) showed that the lack of appropriate organizational structure in the EDOs is one of their activity challenges. This study also suggested efforts towards determining the position and structure of EDOs in the university structure, as well as justifying the roles and responsibilities of EDOs as learning think tanks, as a solution to organizational challenges [3].

Lack of awareness of the organizational mission of EDOs is also considered a challenge in organizational structure. Changiz et al. (2013) showed by examining the expectations of faculty members from EDOs that participants expressed their expectations by committing to the main mission of EDOs [2]. In explaining this issue, we can refer to the key role of organizational culture compared to personal characteristics, which have been emphasized in organizational behavior theories.

Cognitive challenges are also among the other challenges identified in this study. So far, progress has been made in the mental and attitudinal dimensions of EDOs in universities. However, to respond to the evolving needs and keep up with the latest advances in medical education science, efforts must be made to develop useful solutions to reduce cognitive challenges.

If development offices are supposed to focus on improving the quality of education through faculty development, they must have sufficient knowledge in the specialized field of education. However, our results revealed that the educational knowledge of the members of the EDOs is not enough. The main reason for this can be attributed to the fundamental difference between the two categories of sciences. Faculty members in EDOs, especially clinical ones, have studied subjects that, in terms of their nature, fall into the category of science. However, the nature of education has its roots in knowledge, which falls into the category of humanities.

Changiz et al. (2013) revealed the first and most prominent expectation that faculty members expressed from EDOs was their educational role [2]. Therefore, it is clear that the primary requirement for fulfilling this role is having the necessary knowledge in the field of learning and education. Our findings are consistent with Dehnavieh et al. (2019) who listed issues related to knowledge as one of the main subgroups of challenges in EDOs. This study emphasizes training and familiarizing managers and employees of EDOs with medical education concepts [3]. Since managers of EDOs are mostly members of clinical faculty, the necessary transfer of academic knowledge in the field of learning and education has not been provided to these members. On the other hand, from the perspective of the philosophy of science, what a member of the medical university’s faculty has learned in university differs from the nature of medical education and teaching. Therefore, at the beginning of entering into activities in EDOs, educational concepts faculty members may seem strange, unfamiliar, difficult, and incomprehensible.

Communication challenges are among the other challenges of EDOs. The lack of effective communication between managers of EDOs and their colleagues in other schools, as well as with the corresponding colleague in the EDC, and other professional entities, is one of the main challenges in this area.

According to organizational communication theories, human relationships, especially in environments that involve more interactions with people, such as educational settings, are of special importance [26]. Since EDOs deal with a wide range of students, faculty members, educational content, and learning environments, they cannot fulfill their duties effectively without having meaningful communication.

Dehnavieh et al. (2019) mentioned communication as a major challenge for EDOs. In this study, inappropriate intra-university interactions were identified as a challenge. Solutions such as interaction between managers of EDOs and manager of EDC, strengthening communication between EDOs and faculty members and educational departments, providing an appropriate atmosphere to enhance the status of education, holding periodic meetings for coordination of activities, emphasizing more on the penetration of development activities into educational departments, and exchanging experiences between universities were highlighted [3]. In explaining this result, we refer to organizational communication theories. The development of the science of organizational communication, along with the development of industrial psychology, social psychology, organizational behavior, and administrative sciences, has led to the formation of common theories and concepts that have been introduced by organizational communication theorists, mainly by experts in these fields. Unlike classical organizational communication theorists, contemporary organizational communication theorists recognize constructs such as participation, coordination, social networks, and information processing. According to Widyanti (2020), organizational communication is a type of information exchange that provides the basis for perception and emotion between two or more members or a group in the organization and causes organizational networks to take shape to perform organizational tasks [27].

Indeed, our results showed that motivational challenges are among the important challenges of EDOs. Motivation is one of the influential factors in the activity of any organization. The more time-consuming, difficult, or less enjoyable an activity is, the more prominent the role of motivation becomes. Activities related to EDOs are mainly intellectual and sometimes accompanied by operational activities that are necessary for educational advancement, but these operational activities are not desirable for faculty members. On the other hand, these activities are time-consuming and from the perspective of some managers, they may have fewer tangible measurable outputs. Therefore, in these activities, the presence of a motivating factor is of greater importance.

Having a reliable and appropriate reward system with specific plans is important, although creating motivation among employees is not solely dependent on this factor [28]. Consistent with the findings of this study, Dehnavieh et al. (2019) suggested reducing mandatory educational units as a motivating factor for managers of EDOs [3]. Alimohammadi et al. (2021) revealed that individuals differ from each other in terms of motivational dynamics [29]. This point should be taken into account in creating motivational solutions for employees of EDOs. Also, performance evaluation and reward/punishment payment for employees based solely on traditional methods have little effect on improving efficiency and productivity [29]. Sohrabi et al. (2015) showed that an unspecified reward system and irrelevant motivational programs for performance are obstacles to efficiency in government organizations [24].

Our findings showed that motivation is one of the effective factors in the activity of an organization and having a suitable reward system with specific plans is important. It should be noted that individuals differ from each other in terms of motivational dynamics, and this point should be taken into account in creating motivational solutions for employees of EDOs. Also, an unspecified reward system and irrelevant motivational programs for performance are obstacles to improving efficiency in organizations.

Some limitations of this research should be considered. In this study, we only examined the opinions of managers of EDOs, employees of EDOs, faculty members with experience in EDOs, and managers of EDCs in other medical sciences universities. However, it seems that examining the views of other stakeholders such as faculty members, talented students, and educational managers of the university would be useful for enriching the data. Therefore, it is recommended to consider the opinions of other stakeholders in future research. Additionally, this study was conducted only in one medical university, which may negatively affect the generalizability of the results. Therefore, it is suggested that the challenges of EDOs in other medical sciences universities also be examined in future studies.

## Conclusion

Education development offices are one of the main policymaking for quality education in medical universities, but they face challenges in their activities. These challenges were classified into different main categories in this study, and corresponding solutions were suggested. Considering that the managers of these offices are faculty members, these challenges should be addressed specifically, especially in planning to address organizational, educational, motivational, and communication challenges faced by these offices. To address the challenges of organizational EDOs’ activities, efforts are being made to establish EDOs structures within the university and to conduct training courses. The establishment of a clear and performance-based reward system for faculty members who are the managers of the EDOs is proposed. Strengthening interactions between EDOs and faculty members, departments, and the EDC, organizing sessions to coordinate activities, exchange of experiences between universities are highlighted.

## Data Availability

The datasets used and/or analyzed during the current study are available from the corresponding author on reasonable request.

## Abbreviations

EDC: Education Development Center
EDO: Education Development Office
